# A study on occupational health and safety

**DOI:** 10.1186/s12889-022-14584-w

**Published:** 2022-11-25

**Authors:** Lídia Maria Costa Araújo Magalhães, Ketyllem Tayanne da Silva Costa, Gustavo Nepomuceno Capistrano, Maryanna Damasceno Leal, Fábia Barbosa de Andrade

**Affiliations:** 1 Nurse. Master in Public Health, Federal University of Rio Grande Do Norte, Natal, Brazil; 2 Nursing Student. Federal University of Rio Grande Do Norte, National Council Scientific and Technological Development (CNPq), Natal, Brazil; 3 Nursing Student, Federal University of Rio Grande Do Norte, Natal, Brazil; 4 Nurse. Doctor in Health Sciences. Post Graduate Program. Federal University of Rio Grande Do Norte, Natal, Brazil

**Keywords:** Occupational health, Occupational health services, Surveillance, Workers health

## Abstract

**Background:**

This study aimed to evaluate and describe the indicators of occupational health, with a focus on the medical expertise and periodic medical examination.

**Methods:**

This is exploratory-descriptive, cross-sectional, documentary, quantitative, and retrospective research, in the historical series: 2011 to 2015.

**Results:**

The number of lost days of work per worker and the frequency of licenses increased despite the decrease in the Absenteeism Duration Index and stabilization of the Frequency of Medical Workers. As for the adhesion of the workers to the Periodic Medical Examinations, it was decreasing, with a higher percentage in the year 2012 (35.3%). During the analyzed period, 5,186 workers performed the Periodic Medical Examination, and the majority (60.6%) presented non-ideal weight, 41.1% were sedentary, 33.2% had dyslipidemia, 29.0% were alcoholic, 3.2% were smokers, 5.9% had diabetics, and 16.4% reported high noise in the workplace, 27.8% inadequate lighting and 35.9% inadequate work furniture.

**Conclusions:**

The results highlight the need to maintain and strengthen the Worker Health and Safety Policy with emphasis on surveillance, aiming at the promotion and protection of the health of the workers, based on the elaboration of the epidemiological profile of health and, consequently, the implementation of positive impact strategies.

## Introduction

Historically, in Brazil, Occupational Health and Safety (OHS) is strongly associated with the political-social and economic evolution of the country and is presented as the achievement of rights resulting from claims and struggles of the workers. Work is one of the determinants that most impact man’s conditions, quality of life, and health.

Working is essential for human beings since it is the way in which respect, integration, sociability, recognition, and bonds of friendship are obtained. On the other hand, the living conditions of Brazilian workers are aggravated by the alternation of stages of growth and accelerated industrialization with moments of recession, resulting in the government’s adoption of adjustment measures and financial cuts in social policies, such as education, health, safety, transportation, housing, and work, among others [1, 2].

Nowadays, the epidemiological profile of workers' morbidity and mortality in Brazil is characterized by the coexistence of diseases that have an intrinsic relationship with working conditions: diseases related to work and typical work accidents, which have their frequency, appearance, and severity modified by the activity. Added to this reality are diseases common to the population as a whole, which have no etiological relationship with work [3].

Health Promotion and Surveillance refer to the pillar of the Occupational Safety and Health Care Policy (PASS, in Portuguese) that encompasses quality of life and vigilance actions in the environmental and work processes. Standing out among these are the institution of guidelines and programs in the area of mental health and occupational diseases of higher prevalence; the mandatory provision of Periodic Medical Examinations (PME) for all employees; the training in health and safety at work; the creation of an Internal Committee on Health and Safety at Work and a survey of environmental risks, with a stimulus to the active participation of employees in processes involving their health [4, 5].

The PME aims, mainly, the prevention, enabling the health surveillance of the employees of a certain company or institution, contributing to the early identification of diseases related or not to work. It is carried out by an occupational doctor and employers must provide examinations for employees at specific times such as dismissal, admission, leaves of absence or change of function, in addition to periodic examinations, which will vary in frequency according to the workers' age (every two years for those between 18 and 45 years old and annually for those outside this age group) [6].

The PME is performed through clinical examinations, anamnesis, general and specific laboratory tests, according to the function developed by each worker. In addition, the occupational physician must adapt the exams to the particularities of each case, for example, people with disabilities or people who work with noise and may experience deafness caused by this fact. The result of the PME is not given by score or a question of approve or disapprove workers, it is related to the early diagnosis of health problems [6].

It is noteworthy that the information generated during the expertise act are important indicators of worker's health, privileged instruments for the construction of the morbidity and mortality profile of public servants, which will help to conduct the development of health promotion actions, since the expert databases issue a variety of data on the most prevalent diseases and the professionals who get sick [7].

It is of great importance to deepen the study in relation to the health of the federal public servant, considering the need to research, know and analyze the determining and conditioning factors of health problems related to processes and work environments. In this way, it is important to analyze workers' health indicators, which are reflections of the real health conditions of the server, with the objective of guiding managers in the planning and control of activities, in addition to allowing deductions regarding the effects of decisions and their results.

From this perspective, this study aimed to evaluate and describe occupational health indicators focusing on the Official Health Expertise and PME of federal public servants, including professors from the institution and administrative technicians from the education sector of the Federal University of Rio Grande do Norte.

## Materials and methods

This is a cross-sectional, retrospective study with a quantitative approach, where secondary data were obtained regarding PME and official health expertise, specifically the SIAPE HEALTH module of federal public servants of a public institution of higher education in Brazil.

The information contained in this system is federal level and is entered by the experts who perform the exams, uploading them directly into the system, enabling access to the information by users. For the study, secondary and aggregated data from the SIAPE SAÚDE system database were evaluated, as well as management reports made available by the SIASS Unit (Subsistema Integrado de Atenção à Saúde do Servidor) from UFRN, responsible for storing such data.

The study was carried out at the Federal University of Rio Grande do Norte, Central Campus, especially at the Directorate of Attention to Servant Health (DAS), where the SIASS Unit is located, the latter being responsible for coordinating actions in attention to the health of the institution's servants, specifically, the performance of the Periodic Medical Examination and the Official Health Survey, objects of this study.

The period chosen for the study was from 2011 to 2015. The preference for this time interval was justified by the fact that the year 2011 marks the beginning of the PME through the computerized system SIAPE HEALTH, and the end of the study period in 2015 characterizes five complete years and the historical nature of epidemiological studies.

The population chosen for the study can be divided into levels of education, the teachers, technical-administrative in education, higher level positions are level E, while the technical-administrative in education, middle and basic level positions are levels C and D.

The official health expertise and the PME were used as a dependent variable. For each indicator, independent variables were selected: a) Official Health Expertise: gender, age, position, number of active statutory employees away, number of days of leave and number of days away; and b) PME: Gender, age, position, ethnicity, smoking, physical activity, BMI, hypertension, diabetes mellitus, dyslipidemia, spinal pain, inadequate furniture, inadequate lighting, likes what you do, good relationship with the boss, good relationship with colleagues and fast pace. In addition, the following indicators were observed: Absence Severity Index (IGA), Medical Frequency Leave (FML), Frequency of Workers on Sick Leave (FWML) and Absenteeism Duration Index (IDA), as recommended by the Permanent Commission and International Association on Occupational Health [8] and the authors Hensing et al. [9].

The information was obtained from Microsoft Excel spreadsheets, being possible to organize and sort the variables into categories. Then, the data were exported and analyzed in the software Statistical Package for the Social Science (SPSS). Relative and absolute frequency distribution was used for categorical variables, as well as measures of central tendency (average), measures of dispersion (standard deviation), and student's t-test for quantitative variables.

For data analysis, the chi-square test and the calculation of the odds ratio were used for correlation of the indicators, adopting a confidence interval of 95% and a significance level of 5% (*p* < 0.05) for all tests.

Concerning the ethical aspects, the project was submitted to the Research Ethics Committee of Federal University of Rio Grande do Norte where it was appraised and subsequently approved under opinion no. 1.707.691, from the principles of ethical and legal aspects that govern scientific research on human beings, as recommended by Resolution no. 466/12 [10], and the principles expressed in the Declaration of Helsinki.

## Results

The results showed that there were 4,293 (35%) departures from administrative records and 7,946 (65%) absences from work granted by expert examination.

This expert examination is a procedure carried out by a medical expert, whose function is to identify if there is the presence of an illness or to identify if there has been an accident that has made you totally or partially, temporarily, or permanently unable to perform your professional activities [11]. The magnitude of these absences can be portrayed when we calculate the sum of lost work time over the five years, which generated 179,916 days of absenteeism due to illness.

Data regarding the sociodemographic characteristics of the studied population revealed that 67.9% (8,312) of the departures occurred in female workers and, for males, 32.1% (3,927). Regarding the age group, 34.6% (4,234) of the licenses were approved for workers between 51 and 60 years old, 24.0% (2,934) from 41 to 50 years, 19.2% (2,355) from 31 to 40 years, 11.8% (1,449) from 18 to 30 years, and 10.4% (1,267) over 60 years.

In relation to the post variable, the number of workers occupying the position of administrative technician in education levels C and D predominated, with a prevalence of 62.2% (4,941), while 23.8% (1,889) workers were in higher-level positions.

**Fig. 1:**
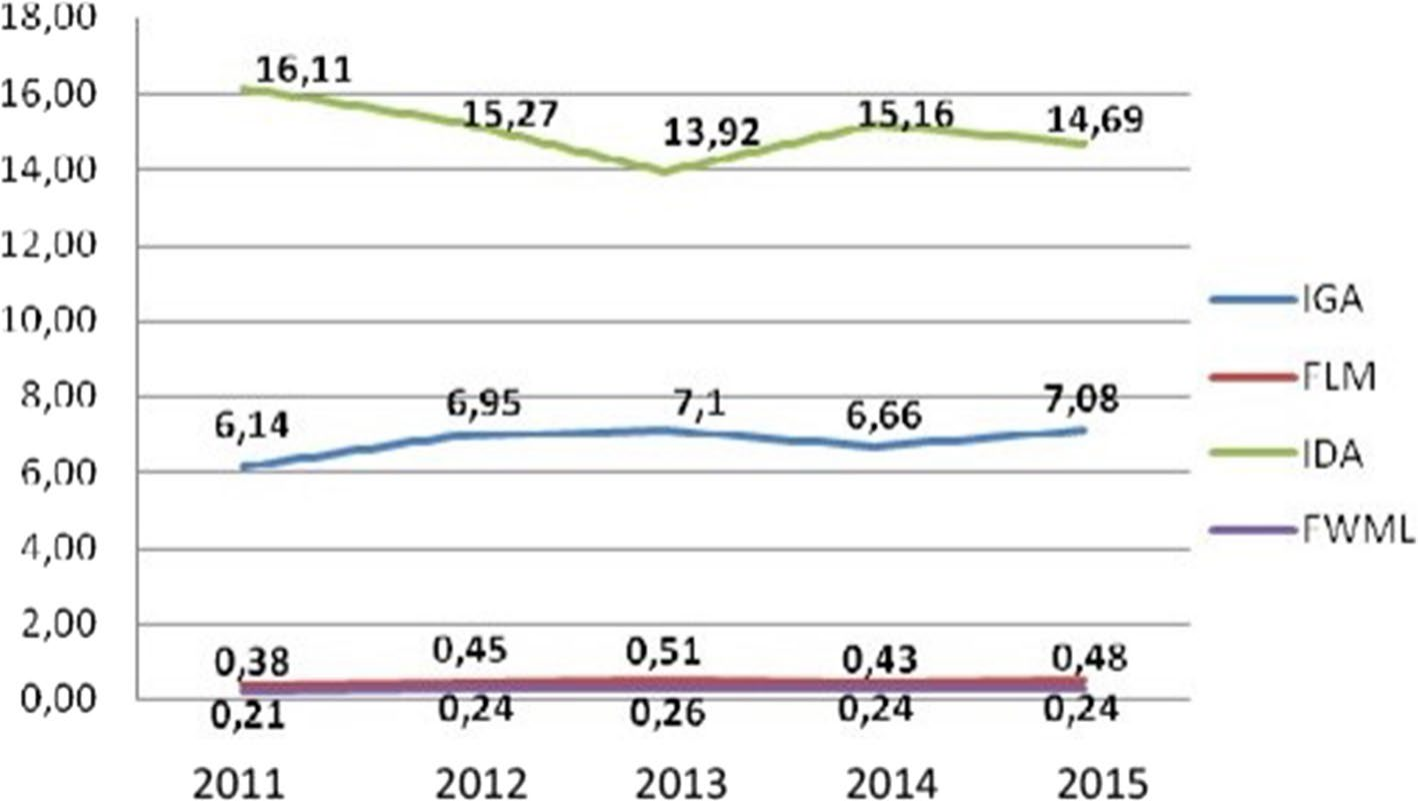
Indicators of absenteeism, 2011–2015. Natal/RN, Brazel, 2017. Legend IGA = Absenteeism Severity Index; FLM = Frequency of Medical Licence; IDA = Absenteeism Duration Index; FTLM = Frequency of Workers on Medical Licence. Source: Elaborated by the Authors

Figure 1 shows the absence of workers at work due to health care in the period from 2011 to 2015. It is noteworthy that there is an increase between 2011 and 2013, when there is a peak of 7.1 days not worked. The following years show an oscillation, but with a tendency for growth.

**Fig. 2:**
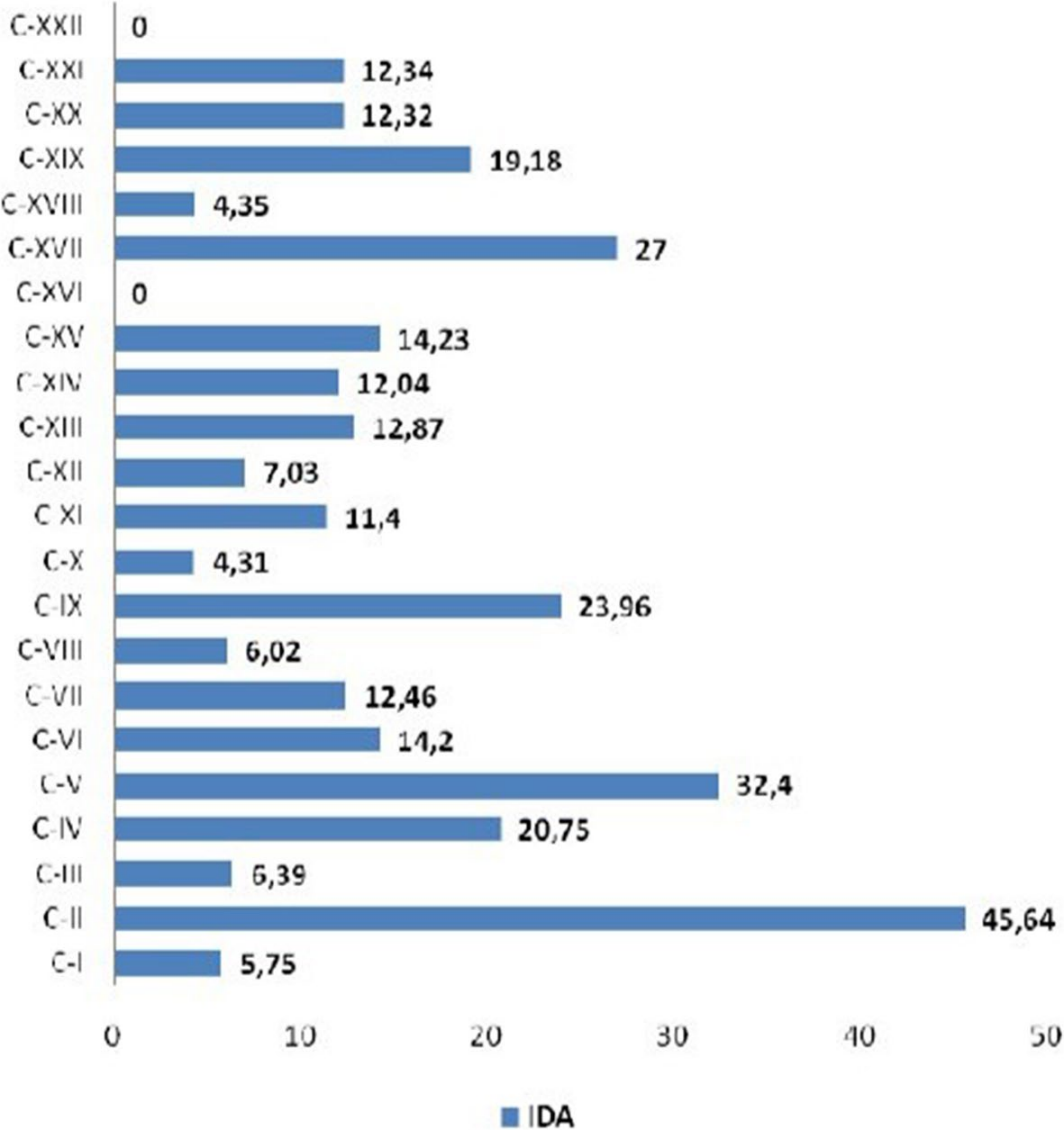
Distribution of IDA, 2011–2015. Natal/RN, Brazil, 2017. Legend: C = ICD.10 chapter. Source: Elaborated by the author

In this sense, it is also relevant to present the individual absence duration, according to the cause of illness, in order to facilitate the adoption of specific measures focused on the pathologies with the greatest impact on lost days of work. Figure 2 shows the IDA according to each International Classification of Diseases (ICD), 10 chapter, and the highest indexes refer to neoplasms (45.64), mental disorders (32.40), congenital malformations (27.00), and diseases of the circulatory system (23.96), respectively. These findings reveal that absences of longer duration were caused by pathologies of a chronic non-transmissible nature, except for causes of absences in chapter XVII of ICD-10.

**Fig. 3:**
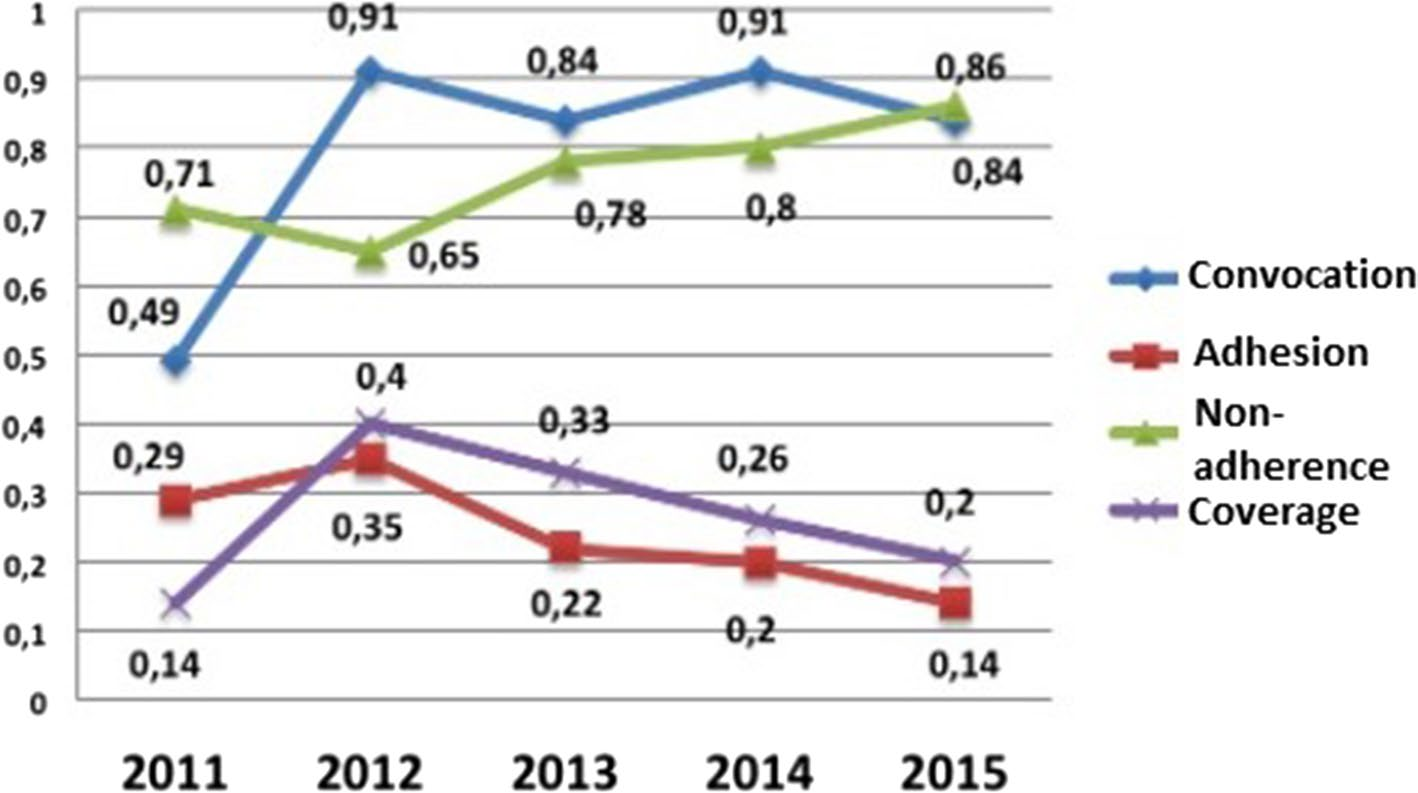
Distribution of call, adhesion, non-adhesion, and coverage ratio to PME, 2011–2015. Natal/RN, Brazil, 2017. Source: Elaborated by the authors

Figure 3 presents the results of this study regarding the adherence of the workers to the Periodic Medical Exam (PME), considering the historical series from 2011 to 2015, when an average of 4,362 workers were called.

The Periodic Medical Exam consists of the periodic clinical and laboratorial evaluation of the worker, due to the existing risks in the work environment and occupational or professional diseases. The PME foresees the adoption of prevention, tracking, and early diagnosis measures for work-related diseases, besides those more prevalent in the general population, such as diabetes mellitus, hypertension, neoplasms, dyslipidemias, and ophthalmologic diseases. Also, the PME will be carried out during working hours, without any burden or need for compensating schedules on the part of the employees. It is important to point out that absenteeism is taken into consideration only due to the worker's personal illness, and this diagnosis cannot be related to someone in the employee's care.

Regarding the operationalization for the PME, it is important to mention that at the moment the server is called for the evaluation of occupational health, through personal e-mail, he/she must fill out the consent form as a way to prove the agreement to participate in periodic medical examinations. Thus, going from the situation "INVITED" to "CONFIRMED". It is worth pointing out the importance and potential of the PME, once it allows the early identification of risk factors for getting sick, as well as the construction of collective diagnoses in the Worker's Health area, which makes this action a health management instrument, for monitoring the health situation and work conditions, and the subsidies for interventions to improve the quality of life of the workers.

It can be observed that the call-up ratio increased by 42.0% from 2011 (0.49) to 2012 (0.91). From the year 2013 (0.84), there were oscillations characterized by drop and growth in the calls.

As for non-adherence, in 2012, there was a decrease, and in 2013 (0.78), 2014 (0.8), and 2015 (0.86), there was an increase in the results, characterizing a relevant increase of 15. 0% between the years of 2011 (0.71) and 2015 (0.86).

About PME membership, it is clear that growth occurred only in 2012 (0.35). Then, the index decreased throughout the series, namely: 2013 (0.22), 2014 (0.2) and 2015 (0.14), which explains the non-adherence data, that comprehends the number of called servers that didn't do the PME in the analyzed year, having as reference the total number of UFRN's servers summoned in the evaluated year as being an unfavorable reality in relation to the PME recommendation. This may be related to factors such as excessive work activities of workers, periodic examinations performed through private health insurance, and to the lack of recognition of the importance of PME by workers.

The coverage ratio of the PME represents the servers that have concluded the PME and those that have an updated Occupational Health Certificate in the analyzed year, with the total number of servers at UFRN in the analyzed period as a reference. This coverage ratio increased significantly in the year 2012 (0.4), showing a growth of 26.0% in relation to 2011. Thereafter, the ratio decreased, with an average of 0.26 between the years of 2013 (0.33), 2014 (0.26) and 2015 (0.2), as shown in Fig. 3.

In the list of risk factors, health indicators of different epidemiological natures were analyzed. Among them, those related to cardiovascular diseases and occupational risk factors, such as the existence of inadequate work furniture and accelerated work rhythm, are presented in Tables 1 and 2.

**Table 1:** Risk factors associated with hypertension, 2011–2015. Natal/RN, Brazil, 2017

	**Total (*n* = 5186)**	**Frequency (%)**	**Raw OR (IC95%)**	**p**
**BMI**				
Non-ideal weight	3143	60.6	0.015 (0.011 – 0.019)	< 0.001
Ideal Weight	2042	39.4		
**Sedentarism**				
Sedentary	2134	41.1	0;001 (0.001 – 0.002)	< 0.001
Non-sedentary	3044	58.7		
**Etilism**				
Yes	1504	29.0	0.646 (0.631 – 0.662)	< 0.001
No	3670	70.8		
**Smoking**				
Yes	167	3.5	0.740 (0.728 – 0.752)	< 0.001
No	5005	96.5		
**Diabetes**				
Yes	305	5.9	1.085 (1.075 – 1.095)	< 0.001
No	4870	93.9		
**Dyslipidemia**				
Yes	1722	33.2	1.801 (1.751 – 1.853)	< 0.001
No	3453	66.6		

*OR* Odds ratio, *p* P value

**Table 2:** Risk factors associated with fast pace of work, 2011–2015, Natal/RN, Brazil, 2017

	**Total (*n* = 5186)**	**Frequency (%)**	**Raw OR (IC95%)**	**p**
**Good relationship with colleagues**				
Yes	4994	96.3	0.764 (0.571 – 1.022)	0.080
No	190	3.7		
**Good relationship with bosses**				
Yes	4957	95.6	0.724 (0.555 – 0.945)	0.021
No	227	4.4		
**Inadequate furniture**				
Yes	1862	35.9	1.558 (1.387 – 1.750)	< 0.001
No	3115	60.1		
**Loud Noise**				
Yes	852	16.4	1.994 (1.719- 2.313)	< 0.001
No	4332	83.5		
**Inadequate lighting**				
Yes	1442	27.8	1.659 (1.466 – 1.877)	< 0.001
No	3536	68.2		
**Likes what do**				
Yes	5055	97.5	1.040 (0.725 – 1.490)	0.856
No	129	2.5		

*OR* Odds ratio, *p* P value

In the list of chronic pathologies covering categories II and III of the Schilling classification, the most common causes of morbidity among workers are: Systemic Arterial Hypertension (SAH), chronic respiratory diseases, diseases of the locomotor system and mental disorders. These are pathologies of multiple etiology in which work is considered a risk factor associated with the increased probability of occurrence of these diseases [12]. Thus, the present study highlights cardiovascular diseases, especially SAH.

Table 1 shows the distribution of the aforementioned risk factors associated with SAH. It is pointed out that 60.6% (3,143) of the workers that performed the EMP presented non-ideal weight; 58.7% (3,044) practiced some type of physical activity; 70.8% (3,670) denied alcohol use; 96.5% (5.005) did not smoke; 93.9% (4,870) did not have diabetes mellitus (DM); and 66.6% (3,453) did not have dyslipidemia. The association between hypertension and all correlated variables was significant at *p* < 0.001. As for the Odds Ratio calculation, we considered the hypertension disease in relation to the following variables: BMI, sedentary lifestyle, alcoholism, smoking, diabetes, and dyslipidemia. The OR calculation does not imply a cause-and-effect relationship, it only suggests that there is an association.

In Table 2, it is possible to observe that 35.9% of the interviewed workers are not adequate for their activities. In addition, 16.4% report loud noise in the workplace and 27.8% do not have adequate lighting. Social factors were also obtained, noting that 3.7% of the workers surveyed say they do not have a good relationship with their co-workers, while 4.4% do not have a good relationship with their boss and 2.5% show dissatisfaction with what they are doing.

## Discussion

It should be noted that absenteeism is a term used to denote the employee's absence from work [13]. The International Organization of Work (OIT) defines it as the period of absence of work that is accepted as attributable to an incapacity of the individual, except for that derived from normal pregnancy or prison [14].

According to the report of the National Audit Office [15], in the city of Guernsey, United Kingdom, approximately 3.8% of working time was lost due to illness, and civil workers became sick for an average of 8.7 days in 2005. In Chile, health workers belong to the category that has the highest rates of disability due to illness, with 14.3 days of absence per worker per year; unlike the university workers, who present 6 days of work lost per year, similar to the results of this research [16]. These findings highlight the data shown in Fig. 1.

Studies found an average of 7.5 lost days of work per year per worker in the nursing area of a university hospital in Brazil [17]. Santos and Mattos [18] observed 9.3 days of absenteeism due to disease for each municipal worker of the city of Porto Alegre in 2005. The studies reported 9.1 and 10.3 days of absence due to illness for each public worker of the municipalities of Goiânia and São Paulo, respectively [19, 20].

The worker and financial conditions can cause work accidents and environmental conditions, increase work capacity and the market, which may exclude work and consumption capacity. The employee is also hit with productivity, lack of manpower, loss of manpower and/or equipment damage [21].

The World Health Organization (WHO) estimates about 36 million annual deaths from Chronic Non-Communicable Diseases (NCDs), composed mainly of circulatory diseases, neoplasms, chronic respiratory diseases and Diabetes Mellitus (DM), which have risk factors.—smoking, alcohol, physical inactivity, unhealthy diet and obesity—modifiable in common [22, 23].

An important characteristic of epidemiological patterns in Brazil concerns the changes in the composition of morbidity and mortality by groups of causes. Thus, the high prevalence of deaths from infectious and parasitic diseases, present at the beginning of the twentieth century, gave way to NCDs and injuries related to accidents and violence [24].

In Brazil, according to the Ministry of Health [23], NCDs are among the main causes of hospital admissions, and the financial cost to the Unified Health System (SUS) represents a growing impact. Estimates for Brazil suggest that the loss of productivity at work and the decrease in family income resulting from chronic pathologies such as diabetes, heart disease and stroke involved spending of US$ 4.18 billion between 2006 and 2015 [25].

The researchers Moura, Carvalho and Silva (2007) [26] carried out a study on the repercussion of CNCDs in the granting of social security benefits by the National Institute of Social Security (INSS) and identified musculoskeletal and circulatory system diseases as the main causes for granting sick pay.

This reality is also revealed among public servants in several studies that present the main groups of causes of sick leave for this category of workers, with high rates of absenteeism due to diseases of the musculoskeletal system and connective tissue, mental and behavioral disorders, chronic respiratory diseases and circulatory system diseases [7, 19, 27–33].

The implementation of strategies to reduce absenteeism is a great challenge for employers, and it is necessary to analyze the events in the workplace to delineate situational diagnoses and guarantee actions to promote worker health. For the authors, the change in the epidemiological profile of illness and the increase in the prevalence of chronic diseases, as shown in Fig. 2, reveal concern for the global scenario regarding the impact of these diseases on workers' health, due to the growth in the number of lost workdays [21].

The epidemiological profile of morbidity and mortality in Brazilian workers is characterized by the coexistence of diseases that have an intrinsic relation with working conditions, and in addition, diseases common to the population are observed, which are not etiologically related to the work [3]. In this reality, it is important to emphasize the importance of the employees performing the Periodic Medical Examination (PME), for the prevention and/or possible early detection of the pathologies that generate the greatest impact on the lost days of work, highlighting the neoplasms [22].

The importance of performing the PME in the screening of risk factors for chronic non-communicable diseases, such as dyslipidemia, sedentary lifestyle, obesity, arterial hypertension, diabetes mellitus, alcoholism, and smoking is highlighted. In addition, through the PME, the workers will be guided and sent to participate in the various health promotion programs offered by the institution. Through these strategies, it is possible to reduce the prevalence of diseases of the circulatory system, another important cause of absenteeism, as shown in Fig. 2.

As for Fig. 3, which shows data on the PME, despite weaknesses, it is evident that the most satisfactory results of PME adherence occurred in the year 2012, a time when workers composed the Integrated Subsystem Unit (SIASS in Portuguese), as well as the constant discussion in forums, national meetings, and events related to the PASS, in a context of articulation in defense of the strengthening of the actions of attention to workers' health, which may have contributed to the results [23–25].

On the other hand, the situational diagnosis of low PME adherence throughout the historical series was possibly influenced by the recent history of PASS construction and the negative impact of the lack of structuring, planning, and evaluation of the actions. Plus, the largest investments and training, by the Ministry of Planning of Brazil, were related to the expert area which reflects as the main activity of the PASS [4].

The implementation of actions of health surveillance and promotion are major challenges for the consolidation of SIASS, since it is still a recent practice to promote health in public sector workplaces. It is necessary to elaborate indicators to support the actions and allow the evaluation of the results, considering that the information generated through indicators consolidates the control and planning of the organizational processes, as well as supports the decision making [25, 26].

This is a prevention tool that has been implemented in Brazil with workers from federal agencies to identify risk factors associated with future illnesses. This approach in the federal public service has had an impact on the quality of preventive health, avoiding the removal of workers from their workplace for a cause classified as a possible prevention of this disease. Another aspect is the increasing number of absences that have been occurring in recent years, that is, the numbers of absenteeism due to physical and mental illnesses, a fact that occurs at increasingly younger workers' ages, which reveals the need for special attention and protector follow-up in their quality of life.

The results presented in this study deserve attention and can contribute to discussions between the professionals of the technical team and managers of the SIASS Unit and PROGESP/UFRN, as it is believed that the production of knowledge about the subject under study can provide the University with instruments, as well as other institutions at the federal public service level, through the PME as an indicator for planning and evaluating Occupational Health actions.

Thus, continuous investments in health policies aimed at public servants are suggested, which contributes to the reduction of illness and early retirement, resulting from disability. In this sense, investment in research that allows a better understanding of the relationship between health and work in the public service is also recommended.

It should be noted that this study had some limitations, as the use of self-reported data by employees who completed the PME may underestimate or overestimate the results presented.

## Conclusions

In order to meet the proposed objective, there was the occurrence of neoplasms, mental disorders, and diseases of the circulatory system in terms of duration of absenteeism (IDA), which were the causes of the absences with a longer duration, which ratifies the epidemiological importance and the impact of non-communicable chronic diseases on workers' health. The gravity index of absenteeism revealed that the number of lost days of work per year per worker increased over the historical series, as well as the frequency of absences.

With regard to the epidemiological profile of the employees who underwent the PME throughout the historical series, it was possible to identify a significant prevalence of overweight in the population. The working conditions were considered satisfactory in the perception of the workers. It should be noted that this study presented some limitations, since the use of self-reported data by the workers may underestimate or overestimate the presented results.

Also observed through this study is the need to maintain and strengthen the PASS with emphasis on surveillance, aiming at the promotion and protection of the health of the workers, based on the elaboration of the epidemiological health profile and, consequently, the implementation of strategies of positive impact for OHS.

## Data Availability

The datasets used and/or analysed during the current study available from the corresponding author on reasonable request.

## Abbreviations

ICD: International Classification of Diseases
DM: Diabetes mellitus
FML: Frequency of Medical Licence
FWML: Frequency of Workers on Medical Licence
OHS: Occupational Health and Safety
IDA: Absenteeism Duration Index
IGA: Absence Severity Index
OIT: International Organization of Work
PASS: Occupational Safety and Health Care Policy
PME: Periodic Medical Examinations
SAH: Systemic Arterial Hypertension
SIASS: Integrated Subsystem Unit
SPSS: Statistical Package for Social Science
